# Fluctuation of Water Intake and of Hydration Indices during the Day in a Sample of Healthy Greek Adults

**DOI:** 10.3390/nu11040793

**Published:** 2019-04-06

**Authors:** Adelais Athanasatou, Aikaterini Kandyliari, Olga Malisova, Maria Kapsokefalou

**Affiliations:** Unit of Human Nutrition, Department of Food Science and Human Nutrition, Agricultural University of Athens, 75 Iera Odos Str., 11855 Athens, Greece; dathanasatou@gmail.com (A.A.); kkandyliari@aua.gr (A.K.); olgamalisova@yahoo.gr (O.M.)

**Keywords:** water intake, beverages consumption, hydration indices, spot urine samples, urine fluctuations

## Abstract

Mild dehydration may occur during specific periods of the day because of poor hydration habits and/or limited access to a variety of beverages or foods, for example, in work environments. Measurement of hydration indices in spot or in 24 h urine samples may mask mild dehydration in specific periods of the day. Healthy subjects (*n* = 164; 74 females; age 38 ± 12 years) living in Athens, Greece were enrolled in the study. Subjects recorded their solid food and drink intakes and recorded and collected all urinations for three consecutive days. Water intake was analyzed in 24 h and 6 h periods from wake-up time and scored for variety. Urine hydration indices (osmolality, volume, color, specific gravity) were analyzed in 24 h samples, in morning urine samples and in samples collected in 6 h periods from wake-up time. Fluctuations during the day were significant for the intake of drinking water, hot beverages, milk, fruit and vegetable juices, and alcoholic drinks and for urine osmolality, volume, color, and specific gravity. The urine volume of the first 6 h period after wake-up time (557 ± 231 mL/day) reflects by 76% the 24 h urine collection (1331 ± 144 mL/day). Water intake from all beverages, with the exception of alcoholic beverages, was greater in the first 6h period (morning period) and decreased throughout the day. Hydration indices changed accordingly. The 6 h timed urine sample collected reflects indices in samples collected over 24 h better than any spot urine sample.

## 1. Introduction

Dehydration is the state of body water depletion [1]; a depletion of about 4–6% of body’s fluids is considered mild dehydration [2]. Euhydration reduces the risk of urolithiasis, the incidence of constipation, exercise asthma, hypertonic dehydration in infants, and hyperglycemia in diabetic ketoacidosis. Moreover, it is associated with reduction in urinary tract infections, hypertension, fatal coronary heart disease [3]. Dehydration is associated with a higher risk of venous thromboembolism [4] and dysnatremia [5]. Mild dehydration is linked to disruptions in mood and cognitive performance, e.g., concentration and short memory, increased reaction time, anxiety, moodiness [6], sleepiness, fatigue, and increased confusion [7], thus affecting our productivity at work and quality of life in general.

Although the importance of prevention of mild dehydration is evident, data on water intake in association with hydration status are limited, particularly in free-living conditions. Studies reported that total fluid intake has a strong correlation with urine biomarkers (osmolality, color, specific gravity, volume) [8]. In most protocols employed in studies on hydration, indices are measured in spot urine specimens or in pooled samples of urine collected over a 24 h period [9,10], thus masking potential fluctuations during the day.

A careful evaluation of hydration indices from specimens collected during the day will reveal whether these hypothesized fluctuations actually occur. Moreover, it will offer insights into whether a morning urine sample or a sample of another short period reflects best hydration. Linking information on water intake, variety in beverage intake, and hydration status will elucidate whether poor habits in hydration may lead during the day to time periods of mild dehydration.

We hypothesized that mild dehydration may occur in a transient manner, i.e., only in time intervals during the day because of poor drinking and eating habits or poor accessibility to a variety of fluids or foods, e.g., at work [11]. This information will uncover whether short time periods of mild dehydration may be observed and subsequently will lead to focused advice on hydration schemes.

The objectives of the study presented herein were to measure, in a sample of Greek adults, (a) water intake from all sources per 6 h periods and the variety score in beverage intake and (b) urine hydration indices in 6 h period urine samples collected over a 24 h period.

## 2. Materials and Methods

Subjects were adults aged 20–60 years with approximately equal numbers in each decade of life. Subjects (*n* = 164, age 38 ± 12 years; BMI 24.9 ± 4.7 kg/m^2^; 54.3% males) living in the metropolitan area of Athens were recruited in the framework of the European Hydration Research Study (EHRS) [12] during winter (*n* = 85) and summer (*n* = 79). Exclusion criteria were disease (diabetes insipidus, renal disease, liver disease, gastrointestinal diseases or problems, cardiac or pulmonary diseases, disease that limits mobility including muscle—skeletal diseases, or orthopedic problems), pregnancy, lactation, hypertensive under severe salt restriction, taking drugs that are, or contain, diuretics, phenytoin, lithium, demeclocycline, or amphotericin, and following a high-protein and/or hypocaloric diet. Subjects were rescheduled or omitted if they caught flu (cold) or had fever, vomiting, and/or diarrhea or menstruation during the data collection period. Urinary volumes < 500 mL and creatinine excretion rate (CER) > 3500 mg/day or <350 mg/day suggest inaccurate urine collection [13]. Energy intake was not considered as an exclusion criterion for the study. The recruitment strategy included invitations (a) sent by email to the non-academic and academic personnel, (b) uploaded on social media and published in local newspapers, (c) uploaded on internet sites related to nutrition, (d) distributed in paper at various non-academic places, (e) sent by email to other academic and social work institutions in the greater area, and (f) distributed at any seminar that the research teams were giving. The response rate was approximately 10%. The recruitment strategy was performed in a random sample of the population, and the subjects that responded to the invitations were categorized according to their age and sex.

The scope of the present study is to administrate an observational study of water intake and hydration indices simultaneously, and not to indicate national representative data for water intake and hydration.

The study protocol was developed according to the ethical principles of the Declaration of Helsinki [14] and was approved by the Research Ethics Committee of Agricultural University of Athens (197/27-02-2012). Written informed consent was obtained from all subjects.

### 2.1. Water Intake

A three-day diary (3DD) was used for the detailed recording of the intake of solid foods and beverages for three consecutive days. Before the initiation of the study, a dietitian had two personal interviews with each subject. The first was on day 0, in order to instruct on how to record the category and amount of solid food and/or beverage consumption using appropriate food photographs and preforms. The second was on the last day of the study in order to examine adherence to protocols. Moreover, our dietician gave a telephone call to each of the subjects during their study days and recorded a 24 h dietary recall which was compared with their recording for that day. Subjects recorded their food and beverage intake, time, and place consumed immediately after it happened in order to avoid misreporting. Portion sizes, method of preparation (i.e., fried, baked), serving size, and package information were also recorded. Participants were instructed to maintain their usual physical activity and eating and drinking habits throughout the study; there was no restriction in the category and number of beverages consumed. Water from beverage intake was analyzed in 6 h periods from the wake-up time of each day: Morning (0–6 h from wake-up time); afternoon (6–12 h from wake-up time); evening (12–18 h from wake-up time); night (18–24 h from wake-up time). Water intake from solid food was analyzed with Diet Analysis plus version 6.1 (ESHA Research, Wadsworth Publishing Co Inc, (Salem, OR, USA)).

### 2.2. Urine Hydration Indices

Urine hydration indices were assessed on a daily basis. Subjects were instructed (a) to collect their urine in a plastic container, (b) to weigh each urination using an electronic digital scale (Soehnle Fiesta 65106), (c) to record the weight of urination and the time of collection, and (d) to transfer approximately 10 mL of each urine sample in a numbered tube using single use pipettes. No preservatives were added in urine samples. Subjects stored the urine tubes in a Styrofoam box using ice packs until arrival to the refrigerator. A 24 h reconstituted sample of 10 mL was prepared in the laboratory by pooling these urine samples in a volume proportional addition of each specimen for each day of the study. All the 6 h reconstituted samples were prepared by pooling urine samples collected over the following time-periods: Morning (0–6 h from wake-up time); afternoon (6–12 h from wake-up time); evening (12–18 h from wake-up time); night (18–24 h from wake-up time). In addition, first morning urine (FMU) samples were collected, stored, and analyzed separately. Urine osmolality was measured in duplicate using a freezing-point osmometer (Cryoscopic Osmometer, Osmomat 030, Gonotec, (Berlin, Germany)). Urine color was determined via the eight-point urine color chart developed by Armstrong (1994). Urine specific gravity was measured with a pen refractometer (Master Reftractometer, Atago, cat. No 2771, (Tokyo, Japan)).

### 2.3. Data Analysis

(A) Beverage consumption was grouped in the following categories: (1) Hot beverages (including tea and coffee); (2) milk (including regular, light, and chocolate milk); (3) fruit and vegetable juices (including nectar, fresh, and mix juices); (4) caloric soft drinks; (5) diet soft drinks; (6) alcoholic drinks; (7) drinking water (including tap and bottled); and (8) other beverages (e.g., non-alcoholic beer). Beverage intake was calculated as the sum of the numbers of beverage items from the eight categories. Total water intake was calculated as the sum of the moisture content in solid foods (Diet Analysis plus version 6.1 ((ESHA Research, Wadsworth Publishing Co. Inc., Salem, OR, USA), Database for the traditional Greek recipes [15]) and of beverage intake.

(B) The variety score was calculated as the number of beverage categories, as described above, consumed with a minimum value of “0” and a maximum value of “8”.

(C) Subjects were categorized according to total water intake as low drinkers (≤2.0 L/day for females (*n* = 33); ≤2.5 L/day for males (*n* = 59)) or high drinkers (>2.0 L/day for females (*n* = 41); >2.5 L/day for males (*n* = 31)) using as limits the European Food Safety Authority (EFSA) Dietary Reference Values for adequate intake of water for males and females (2.5 L and 2.0 L, respectively) [2].

### 2.4. Statistical Analysis

Continuous variables are expressed as mean (standard deviation) for variables following normal distribution and as median (P25–P75) for the nonparametric variables. Normal distribution of all continuous variables was tested with the parametric test Shapiro–Wilk or Kormogorov–Smirnov test or graphically assessed by histograms or P–P plots depending on the number of variables. Correlations between normal variables were evaluated using Pearson’s correlation coefficient. Differences between low and high drinkers were performed with student’s *t*-test for normal distribution variables. The Kruskal–Wallis rank–sum test was used to compare the nonparametric variables. Homogeneity was tested with Levene’s test. The multivariate associations between variables were assessed using linear regression models, adjusted for plausible confounders (season, BMI, sex, age, smoking). The potential of each 6h period to estimate the 24 h value was evaluated using the indices of urine osmolality and urine volume, due to their broad physiological ranges. The variation in urine hydration indices and beverage consumption was examined based on the effect of time using mixed linear models, adjusted for relevant biologically plausible confounders. We deemed statistical significance at α = 0.05. Statistical analysis was performed by SPSS package, version 18 (SPSS Inc, Chicago, IL, USA).

## 3. Results

The studied population consisted of 164 Greek subjects; 85 subjects (age 37 ± 13 years; 33 females) completed the protocol during the winter period (December, January, February) and 79 (age 39 ± 12 years; 41 females) during the summer period (June, July, August). The mean BMI of males was 25.5 ± 6.2 kg/m^2^, and that of females 24.0 ± 5.1 kg/m^2^ (*p* = 0.164). In all dietary and urinary variables, no differences were observed (one-way ANOVA) among the 3 days of the experiment (*p* > 0.05); mean values of the 3 days were used to all the analyses.

### 3.1. Fluctuations During the Day

**Figure 1:**
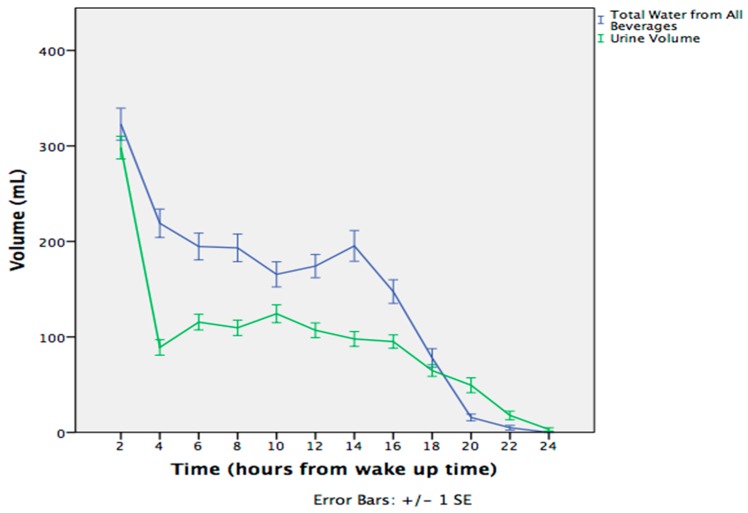
Volume of total water intake from all beverages and of urine excretion during the day.

Significant differences were observed in total water intake from all beverages and urine volume in 6 h periods throughout the day. A peak on the beverage curve was observed in the morning and in the evening 6 h period, while the lowest values were observed at the end of the day. Urine volume was positively correlated with total beverages consumption (*r* = 0.387, *p* < 0.001) and total water intake (*r* = 0.392, *p* < 0.001). Only during the night period was the excreted urine volume greater than the total volume of consumed beverages (Figure 1). This was expected, because the subjects were not consuming any beverages during their sleeping period, resulting only in urine excretions in the morning.

**Figure 2:**
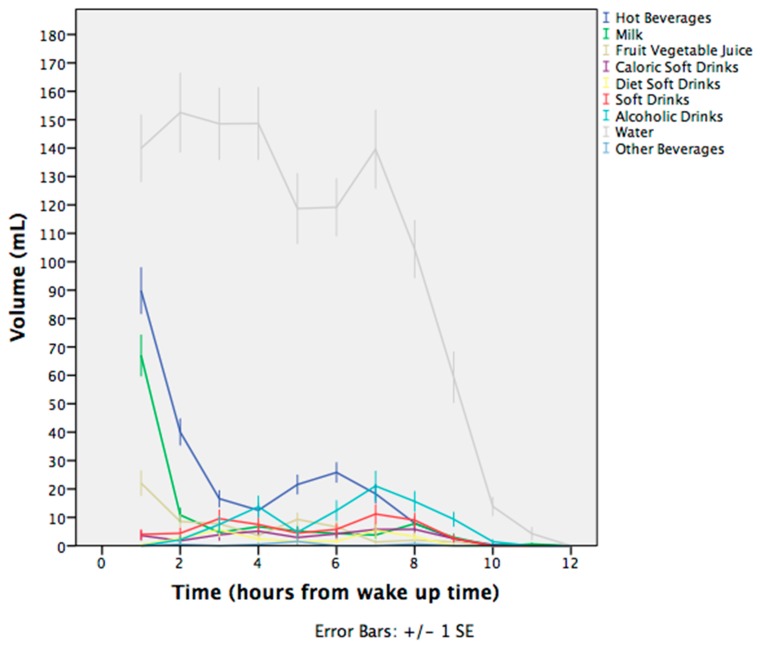
Volume of each category of beverage consumed during the day.

A significant effect of time was present in the intake of water (F = 32.582; *p* < 0.001), hot beverages (F = 90.876; *p* < 0.001), milk (F = 17.063; *p* = 0.003), fruit and vegetable juices (F = 3.785; *p* = 0.045), and alcoholic drinks (F = 7.075; *p* = 0.002) for the 6 h periods. Water was the most preferred beverage and consumed in larger volumes during the day compared to the other seven categories of beverages. A peak in the intake of hot beverages and milk was observed in the morning 6 h period (Figure 2). Moreover, a peak on the curve of alcoholic drinks was observed in the evening 6 h period (Figure 2). The intake of other categories of beverages was spread during the day with no significant differences. Water Intake in the morning interval correlated with urine osmolality in the morning, afternoon, and evening interval (*r* = −0.169, *p* = 0.049, *r* = −0.275, *p* < 0.001; *r* = −0.216, *p* = 0.014 respectively) and Urine Specific Gravity (USG) in the afternoon interval (*r* = −0.260, *p* = 0.003), while water intake in the afternoon interval was correlated with urine osmolality in the afternoon and evening interval (*r* = −0.171, *p* = 0.050; *r* = −0.216, *p* = 0.014) and USG in the evening interval (*r* = −0.231, *p* = 0.008).

### 3.2. Water Intake from Beverages

The mean daily total water intake from beverages was 2266 mL; the range of total water intake was from 470 to 5230 mL/day. The contribution of beverages to total water intake was 1796 (768) mL, while the contribution of foods was 463 (350) mL to total water intake; no differences were observed in water intake from foods between the summer and winter period (*p* > 0.005). In Table 1 are presented the categories of beverages consumed along with their mean volumes (standard deviation) in 6 h periods and 24 h periods. The most popular beverage consumed was water (47%), followed by hot beverages (15%), alcoholic drinks (8%), caloric and diet soft drinks (7% each one), milk (6%), fruit and vegetable juices (6%), and other beverages (4%), with volumes from 10–40% of water intake.

In a 24 h period, significant differences were reported in water (*p* < 0.001), fruit/vegetable juice, and alcohol (*p* < 0.05 for both) consumption between low and high drinkers. Furthermore, a higher total water intake (*p* < 0.001), derived from a higher intake of water (*p* < 0.001) and alcoholic drinks (*p* < 0.05), was observed in the summer compared to winter period of the study. Moreover, total beverage intake was positively correlated with total water intake (*r* = 0.896; *p* < 0.001) and the variety score (*r* = 0.238; *p* < 0.001). Total water intake from beverages was significantly different between subjects that consumed volumes higher than EFSA recommendations (HIGH) and those that consumed lower volumes (LOW) (*p* < 0.001). Differences were also observed in total water intake from beverages and drinking water intake between the summer and winter period (*p* < 0.001).

### 3.3. Urine Hydration Indices

Mean hydration indices (volume, osmolality, specific gravity, color) for 6 h periods, first morning, and 24 h urine samples are presented in Table 2. There was a strong correlation between USG and osmolality (*r* = 0.635), color and osmolality (*r* = 0.716), and USG and color (*r* = 0.553).

The effect of time was significant for all the following urinary hydration indices: Volume (F = 117.191; *p* < 0.001), osmolality (F = 65.228; *p* < 0.001), USG (F = 5.096; *p* = 0.003), and color (F = 65.123; *p* < 0.001). Urine osmolality measured on samples collected in the morning 6 h period and in the first morning urine samples were far more likely to accurately reflect 24 h urine osmolality, compared to evening, afternoon, and night collections. Urine osmolality values obtained from the first morning urine samples can explain the 51% of the 24 h value. In descending order of agreement with 24 h urine osmolality were the morning (48%), night (47%), evening (40%), and afternoon (37%) periods (data not shown in tables). The urine volume of the morning 6 h period samples reflects by 76% the urine excretion over a day (24 h collection) (data not shown in tables). The lowest urine volume was recorded during the night 6 h period. 

Differences were also observed between LOW and HIGH drinkers in the morning, afternoon, evening, and night 6 h periods (Table 2). Hydration indices in first morning urine samples were not different between LOW and HIGH drinkers. A 24 h urine collection showed that LOW drinkers produced decreased urine volumes and had more concentrated samples, as reflected by a higher urine osmolality and a higher specific gravity (*p* < 0.05). The morning 6 h period can explain the 66% of 24 h urine osmolality and 72% of 24 h urine volume of LOW drinkers (data not shown in tables). Values from the afternoon interval can explain the 53% of 24 h osmolality and 54% of 24 h volume of HIGH drinkers (data not shown in tables).

Moreover, differences were observed between the winter and summer period in urine volume in the afternoon (*p* < 0.05) and night (*p* < 0.05) 6 h period samples.

Using cutoffs of 24 h urine osmolality [9,16], 31% of males (*n* = 28) and 23% (*n* = 17) of females were classified as dehydrated, with osmolality values over 800 mOsmol/kg H_2_O observed in most of their short time intervals. A detailed analysis of dehydrated subjects showed that 68% (*n* = 19) of males and 53% (*n* = 9) of females were not complying with EFSA adequate intake.

## 4. Discussion

This study contributes with new data and compares, for the first time, fluctuation in hydration indices and water intake. A series of hydration indices from 6 h interval periods, first morning, and 24 h urine samples collected over three consecutive days were measured in a sample of 164 healthy subjects and compared with water intake from three-day dietary records.

The most important finding in the present study is that fluctuations in water intake occurred during the day, resulting in respective fluctuations in hydration indices. Water intake revealed changes during the day with the highest intake reported in the morning time period and the lowest at the end of the day, both for low and high drinkers. This was also reflected in the urine 6 h period (morning, afternoon, evening, night) volume indices. The volume of morning samples was greater, and it decreased throughout the day. 

The second important finding is that the sample collected during the morning 6 h interval sample may be preferable to the morning spot urine sample in hydration research when 24 h collection is not possible. Samples from 24 h urine collections are considered the “gold standard method” but have increased burden and practical difficulties for subjects [17]. Therefore, spot urine collections have been proposed (spot, timed, daytime, evening, overnight), particularly morning spot urine samples [18], as alternatives with a reduced response rate of participation or errors in adherence to the protocol [19,20]. Subjects that consume more fluids on a daily basis void larger urine volumes [21].

In the study presented herein, hydration indices measured in samples collected from the morning 6 h interval reflect those measured in samples collected over 24 h better than those in morning urine samples. Urine osmolality of the morning 6 h period can explain 66% of the 24 h value of the LOW drinkers group; the afternoon 6 h period can explain 53% in HIGH drinkers. The urine volume of the morning 6 h period could explain 72% and 88% of the 24 h value for LOW and HIGH drinkers, respectively. A previous intervention study [22] with time scheduled water intake reported fluctuations during the day, with larger volumes in afternoon and evening periods. Moreover, the study of Bottin et al. [23] found that 24 h urine concentration can be approximated from a mid- to late-afternoon spot urine sample. It is well documented that first-morning urinations, although often used in population sampling and health-care settings, tend to overestimate 24 h urine concentration [16,22]. First morning urine samples could not detect differences in hydration indices between groups, although differences were observed in 24 h collection. This is maybe due to the fact that the rapid intake of a large volume of water dilutes the plasma and the urine even if dehydration exists [24]. Between LOW and HIGHT drinkers, regarding BMI, there was homogeneity (*p* = 0.74), but there was not regarding age (*p* = 0.26), (data not shown here).

The third important finding is that 31% of males and 23% of females were classified as dehydrated over the day. This was estimated using cutoffs of the 24 h urine osmolality measurements [9,16], with dehydration observed in most of their short time intervals. In the European Hydration Research Study (EHRS), approximately 20% of subjects from three European countries (Spain, Germany, Greece) were dehydrated [12]. Nevertheless, it is believed that chronic dehydration does not occur in healthy, free-living individuals, with ad libitum access to food and beverages [25].

The fourth important finding is that in the studied population, 55% of females and 34% of males followed the recommendations for daily water intake from The European Food Safety Authority (EFSA). EFSA suggests that the adequate intake of water from drinking water, beverages, and solid and fluid foods is 2.5 and 2.0 L/day for males and females [26], although depending on physical activity levels, environmental conditions or health status, higher water intake may be necessary. Recommendations that promote water intake have been issued worldwide by several organizations [27,28,29]. Inter-individual differences among subjects may result in different water intake requirements because of the dynamic complexity inherent in the human water regulatory network [30].

In studies conducted around the world, the percentages for adherence to EFSA recommendations vary. In particular, total water intake measured from 3-day food diaries in a sample of the Spanish population was below the recommendation of EFSA both in men and in women [11]. Data from the “National Diet and Nutrition Survey” in the UK, using dietary weighted records, revealed that 33% of men and 23% of women had inadequate total water intake [31]. On the other hand, the study of Ferreira-Pego et al. [32] showed that 40% of men and 60% of women from 13 countries complied with the EFSA adequate intakes for water intake from fluids. It should be mentioned, though, that offering a wider variety of beverages could increase the daily water intake. However, in our study, subjects had no restrictions on their eating and drinking choices and in the amount and volumes they consumed. This is also confirmed by the fact that pure water and beverages containing water affected the hydration indices in similar way [3].

Additionally, another finding is that the mean total water intake was 2266 (781) mL/day. Previous studies reported total water intake 2270 g/day [31] in UK, 1488 mL/day in China [33], 2.47 L/day in Germany, 1.90 L/day in Spain, and 1.50 L/day in Japan [32]. Water was the most popular beverage of our subjects (47% of total water intake). This finding is in accordance with the study of Armstrong, Johnson, Munoz, Swokla, Le Bellego, Jimenez, Casa, and Maresh [9], in which water was consumed in similar volumes, as well as the study of Perrier, Vergne, Klein, Poupin, Rondeau, Le Bellego, Armstrong, Lang, Stookey, and Tack [10], with water being the major contributor to fluid intake. The study of Bougatsas, et al. [34] showed that drinking patterns, including increased water and milk consumption, were associated with improved hydration, as indicated by lower 24 h urine osmolality. British people consume about 3.3 categories of beverages [31], while the Greek sample of our study 3.6 categories of beverages. In future work, it is of interest to identify dietary patterns that are related with hydration patterns and/or urine and blood indices.

There are some limitations in the present study that should be noted. The 24 h urine collection and food recording has a high burden for the subjects; this may affect their dietary behavior during the experimental period [35]. Moreover, water intake and urine volume were self-reported values and may be subject to participants under- or overreporting. To avoid misreporting, subjects were instructed to record drinks and food ingested immediately after it happened, but some underreporting was conceivable. It must be also mentioned that, because of our recruitment methodology, the sample was not representative to the Greek population; therefore, data may be interpreted with caution. This study is not representing the whole population; however, the correlations between different measurements highlight the availability of different indices to obtain information about hydration.

## 5. Conclusions

In conclusion, fluctuations during the day in water intake and in hydration indices occur. Water intake from all beverages, with the exception of alcoholic beverages, was greater in the first 6 h period (morning period) and decreased throughout the day. Hydration indices changed accordingly. A morning and/or afternoon 6 h period urine sample, and not a spot urine sample (e.g., first morning urine sample) may be an alternative to the 24 h urine collection. This new proposed 6 h timed urine collection protocol could monitor levels of hydration in a daily basis easily, inexpensively, and with reduced burden for participants. Future research in different populations with other cultures and different dietary habits is required in order to proceed towards practical applications of current work.

## Figures and Tables

**Table 1:** Category of beverages consumed in each 6 h period and over a 24 h period for the total sample, LOW drinkers (subjects with a total water intake lower than European Food Safety Authority (EFSA) dietary references), HIGH drinkers (subjects with a total water intake higher than EFSA dietary references), winter and summer period.

Category of Beverage	Morning (A)	Afternoon (B)	Evening (C)	Night (D)	24 h
Hot beverages (mL)					
Total	211 (120)	123 (78)	115 (75)	91 (49)	376 (245)
LOW	197 (101)	112 (79)	83 (78, 129)	88 (42)	360 (232)
HIGH	225 (137)	141 (74)	83 (80, 117)	94 (56)	394 (260)
Winter	200 (124)	123 (85)	83 (82, 142)	95 (55)	379 (269)
Summer	223 (114)	124 (64)	80 (67, 110)	86 (41)	373 (215)
Milk (mL)					
Total	133 (104)	57 (57)	70 (48)	-	160 (123)
LOW	127 (97)	47 (52)	80 (17, 92)	-	146 (109)
HIGH	140 (112)	80 (12, 127)	80 (35, 97)	-	178 (138)
Winter	117 (92)	45 (11, 83)	83 (16, 83)	-	146 (108)
Summer	151 (115)	33 (4, 83)	80 (12, 105)	-	177 (138)
Fruit & Vegetable Juice (mL)					
Total	134 (85)	104 (78)	75 (33, 103)	-	147 (101)
LOW	83 (67, 165)	83 (77, 112)	75 (46, 83)	-	115 (75) *
HIGH	110 (83, 220)	83 (75, 98)	75 (9, 162)	-	176 (115)
Winter	83 (67, 167)	93 (79, 93)	-	-	126 (79)
Summer	137 (82, 217)	83 (75, 113)	-	-	165 (116)
Caloric Soft Drinks (mL)					
Total	110 (50, 158)	110 (83, 167)	133 (83, 167)	-	188 (120)
LOW	110 (33, 167)	110 (83, 167)	117 (50, 187)	-	167 (83, 250)
HIGH	105 (58, 202)	97 (77, 175)	147 (96, 167)	-	167 (83, 267)
Winter	110 (108, 167)	97 (65, 188)	83 (37, 167)		143 (83, 243)
Summer	67 (33, 150)	110 (83, 167)	160 (100, 180)		210 (110, 300)
Diet Soft Drinks (mL)					
Total	167 (75, 230)	110 (88, 153)	110 (77, 167)	-	165 (108, 257)
LOW	138 (75, 167)	167 (167, 167) *	97 (76, 143)	-	167 (100, 235)
HIGH	227 (93, 371)	105 (75, 110)	110 (72, 250)	-	110 (105, 407)
Winter	167 (87,200)	133 (88, 167)	110 (80, 142)		165 (92, 235)
Summer	-	110 (65, 110)	-		167 (110, 540)
Alcoholic Drinks (mL)					
Total	88 (42, 167)	123 (103)	154 (138)	58 (25, 94)	198 (237)
LOW	83 (42, 156)	83 (41, 130)	88 (72, 167)	75 (75, 75)	145 (134) *
HIGH	130 (26, 479)	100 (70, 167)	109 (81, 216)	-	261 (311)
Winter	63 (20, 104)	75 (42, 113)	83 (42, 167)	-	120 (90) *
Summer	145 (80, 271)	110 (67, 193)	122 (83, 220)	58 (25, 94)	266 (298)
Drinking Water (mL)					
Total	465 (290)	414 (247)	345 (272)	83 (83, 162)	1168 (666)
LOW	321 (224) **	326 (198) **	254 (179) **	83 (83, 133)	831 (455) **
HIGH	627 (270)	511 (261)	444 (319)	100(80,167)	1574 (655)
Winter	355 (227) **	337 (205) **	271 (198) **	83 (83, 83)	902 (448) **
Summer	578 (305)	499 (263)	428 (317)	133 (80, 167)	1473 (742)
Other Beverages (mL)					
Total	83 (83, 83)	83 (83, 83)	110	-	110 (83, 167)
LOW	83 (83, 83)	83 (83, 83)	110 (110,110)	-	138 (110, 138)
HIGH	83 (83, 83)	-	-	-	83 (83, 83)
Winter	83 (83, 83)	83 (83, 83)	-	-	138 (110, 138)
Summer	83 (83, 83)	-	-		83 (83, 83)
Total Water Intake (mL)					
Total	863 (425)	750 (357)	533 (333)	22 (64)	2266 (781)
LOW	640 (271) **	602 (278) **	419 (240) **	19 (61)	1564(333) **
HIGH	1147 (416)	938 (359)	679 (377)	26 (67)	2741 (622)
Winter	718 (330) **	642 (325) **	462 (280) **	22 (72)	1942 (527) **
Summer	1019 (461)	865 (355)	610 (367)	23 (53)	2611 (859)
Variety Score					
Total	2.7 (1.1)	2.3 (1.2)	1.8 (1.1)	0.7(0.7)	3.6 (1.4)
LOW	2.6 (1.0) *	2.2 (1.0)	1.8 (1.0)	0.6 (0.6)	3.5 (1.4)
HIGH	2.9 (1.1)	2.3 (1.2)	2.0 (1.0)	0.8 (0.7)	3.7 (1.4)
Winter	2.7 (1.0)	3.7 (1.1)	1.9 (1.1)	0.7 (0.6)	3.7 (1.1)
Summer	2.6 (1.3)	3.5 (1.6)	1.8 (1.1)	0.7 (0.7)	3.5 (1.6)

Results are presented as mean (SD) for normally distributed variables, and as median (P50) and 1st–3rd quartile (P25–P75) for the skewed variables; *p*-values derived through Student’s *t*-test for normally distributed variables and through the Mann–Whitney *U*-test for the skewed variables; * Significant difference between low and high drinkers or between winter and summer (*p* < 0.05), ** Significant difference between low and high drinkers or between winter and summer (*p* < 0.001).

**Table 2:** Urine hydration indices of subjects in each 6 h period, in first morning, and 24 h samples for the total sample, LOW drinkers (subjects with a total water intake lower than EFSA dietary references), HIGH drinkers (subjects with a total water intake higher than EFSA dietary references), winter and summer period.

Hydration Indices	Morning	Afternoon	Evening	Night	First Morning Urine	24 h
Volume (mL)						
Total	557 (231)	378 (205)	290 (158)	177 (149)	339 (123)	1331 (44)
LOW	524 (203) *	353 (200)	272 (146)	174 (160)	327 (109)	1239 (474) *
HIGH	601 (258)	410 (209)	312 (171)	182 (138)	354 (138)	1454 (589)
Winter	578 (212)	413 (206) *	294 (146)	228 (163) *	340 (115)	1397 (472)
Summer	531 (251)	334 (197)	284 (172)	126 (112)	338 (132)	1253 (597)
Osmolality (mOsmol/kg H_2_O)						
Total	620 (240)	627 (258)	580 (254)	271 (157)	691 (224)	665 (223)
LOW	645 (206)	679 (257) *	610 (220)	310 (171) *	719 (225)	703 (209) *
HIGH	588 (278)	558 (243)	540 (291)	221 (123)	655 (219)	620 (233)
Winter	624 (242)	605 (243)	575 (210)	290 (189)	681 (208)	664 (209)
Summer	616 (240)	655 (274)	586 (303)	266 (137)	701 (240)	667 (237)
USG						
Total	1.017 (0.006)	1.017 (0.006)	1.019 (0.007)	1.016 (0.007)	1.018 (0.008)	1.017 (0.006)
LOW	1.018 (0.006)	1.018 (0.006) *	1.020 (0.006) *	1.017 (0.007)	1.019 (0.007)	1.018 (0.006) *
HIGH	1.017 (0.007)	1.016 (0.006)	1.017 (0.007)	1.016 (0.008)	1.017 (0.008)	1.016 (0.006)
Winter	1.017 (0.006)	1.017 (0.006)	1.019 (0.006)	1.014 (0.007) **	1.019 (0.006)	1.017 (0.006)
Summer	1.017 (0.007)	1.018 (0.007)	1.019 (0.008)	1.020 (0.007)	1.017 (0.009)	1.017 (0.006)
Color						
Total	3 (2)	3 (1)	3 (2)	1 (2)	3 (1)	4 (2)
LOW	3 (1)	3 (1)	3 (1)	1 (1)	3 (1)	4 (2)
HIGH	3 (2)	3 (1)	3 (1)	2 (2)	3 (2)	3 (2)
Winter	3 (1)	3 (1) **	3 (1) *	1 (1)	3 (1)	4 (2)
Summer	3 (1)	3 (1)	3 (1)	1 (1)	3 (2)	4 (2)

Values presented as mean (SD); * Significant difference between low and high drinkers or between winter and summer (*p* < 0.05); ** Significant difference between low and high drinkers or between winter and summer (*p* < 0.001). USG: Urine Specific Gravity.
